# Cardiovascular Effects of *Cydonia oblonga* Miller (Quince)

**DOI:** 10.1155/2022/3185442

**Published:** 2022-10-10

**Authors:** Atefeh Amerizadeh, Golnaz Vaseghi, Nazgol Esmaeilian, Sedigheh Asgary

**Affiliations:** ^1^Isfahan Cardiovascular Research Center, Cardiovascular Research Institute, Isfahan University of Medical Sciences, Isfahan, Iran; ^2^Applied Physiology Research Center, Cardiovascular Research Institute, Isfahan University of Medical Sciences, Isfahan, Iran

## Abstract

*Cydonia oblonga* Miller (quince) is a monotypic genus in the Rosaceae family which used to treat or prevent many medical conditions such as diabetes, cancer, infections, and ulcer. This review will summarize the current state of knowledge available on botany, phytochemistry, and pharmacology of this plant with a focus on its effect on some cardiovascular risk factors such as diabetes, blood pressure, lipid profile, and body weight. Databases of Google Scholar, Web of Science, PubMed, Scopus, and SID were searched systematically for English published articles with no date limitation. There were no human studies found, and all of the studies were conducted on animals or *in vitro* models. Reviewing of all 12 included articles showed that different types of quince extract have positive effects on cardiovascular-related factors such as blood pressure, diabetes, glucose metabolism, lipid profile, ROS, body weight, liver dysfunction, and thrombosis. An antihypertensive effect of quince showed to be a magnitude that is comparable to captopril and the lipid-lowering effect of quince showed to be a magnitude that is comparable to simvastatin. However, two studies evaluated the effect of quince fruit extract on insulin levels; one of them reported no positive effect, and the other one reported a significant positive effect. It can be concluded that different parts of quince including leaf, seed, and fruit could be used for improving cardiovascular-related factors including blood pressure, glucose metabolism and diabetes, obesity, and lipid-adjusting purposes. Quince was also found to have strong anti-inflammatory and antioxidant capacity. This study paves the way for further studies on the cardiovascular effect of quince consumption as a beneficial nutraceutical in humans.

## 1. Introduction

Dr. Stephen DeFelice coined the term “Nutraceutical” from “Nutrition” and “Pharmaceutical” in 1989 [1]. Nutraceuticals, in contrast to pharmaceuticals, are substances, which usually have no patent protection. Both pharmaceutical and nutraceutical compounds might be used to cure or prevent diseases, but only pharmaceutical compounds have the governmental sanction. Consumers are turning increasingly toward food supplements to improve well-being when pharmaceuticals fail [2, 3]. Nowadays, nutraceuticals have received considerable interest due to their potential nutritional, safety, and therapeutic effects [4, 5]. According to previous studies, many medicinal plants such as fenugreek, sumac, dill, ginger, grape, and green tea improve the lipid profile significantly [6–10].

Quince is one of the herbal nutraceuticals that has been used throughout olden times [11]. *Cydonia oblonga* Miller (COM) leaves are used in traditional medicines for cardiovascular disease (CVD) treatment or prevention [12]. Its fruit is employed in the food sector as a source of pectin, which defends the colon from damage in IBS and peptic ulcers [13–15]. The plant's seeds have long been used to cure cough, diarrhea, dysentery, constipation, sore throat, and bronchitis [16–18]. Quince extract has been proven to have cancer-fighting, antihemolytic, and antidiabetic properties [19–22]. It is also found to have an anti-inflammatory and antioxidant capacity [23], antiallergic [24], antidepressant, and antistress effect [25, 26]. In addition, the plant contains phenol peroxidase, an enzyme that decolorizes hazardous aromatic colors in industrial wastewater [27, 28]. Quince's widespread therapeutic use and valuable phytochemical composition prompted us to write a review on it, with a focus on its cardiovascular effects.

### 1.1. General Characteristics

The quince (*Cydonia oblonga*), is a nondrought-tolerant small deciduous tree with fruits that look like pears and a superficial and fasciculate root [11]. When exposed to air, the white-yellow pulp becomes hard, or sour, bitter, and acidic, making it unfit for consumption as a fresh product (just a few cultivars are), but when mature, the quince has a pleasant, durable, and powerful flavor. The existence of essential oil in quince fruit, whose odor-active components are considered to be monoterpene lactones and oxides, is responsible for the fruit's strong and distinctive odor [29].

### 1.2. Distribution

The quince fruit is a native of Western Asia and Southwest Europe and has been originated from Armenia, Iran, Azerbaijan, Turkmenistan, and southwest Russia [29]. Turkey produces the most quinces worldwide, ahead of China, Uzbekistan, Morocco, Iran, Azerbaijan, Argentina, and Spain (FAO, 2013).

### 1.3. Taxonomy

The quince is the only member of the genus *Cydonia*, which belongs to the Rosaceae family, subfamily Maloideae, and tribe Pireas. [11, 30].

  Domain: Eukaryota
  Kingdom: Plantae
  Phylum: Spermatophyta
  Subphylum: Angiospermae
  Class: Dicotyledonae
  Subclass: Caryophyllidae
  Order: Rosales
  Tribe: Pireas
  Subfamily: Maloideae
  Family: Rosaceae
  Genus: *Cydonia*.
  Species: *Cydonia oblonga*

### 1.4. Phytochemistry

Different portions of the quince contain phenolic compounds, essential oils, organic acids, ionone glycosides, and tetracyclic sesterterpenes [31–35]. In the pulp, leaves, peel, seeds, and complete fruits of quinces, 27 polyphenolic substances have been discovered including citric, ascorbic, malic, oxalic, quinic, fumaric, and shikimic acids. The sugar profile of the quince fruit includes glucose, fructose, maltose, and sucrose with substantial levels of quinic and malic acids [11, 26, 29]. The fruit contains vitamin C and various minerals such as sodium, calcium, phosphorus, potassium, and nitrogen [36]. COM seeds contain triterpenes, sterols, and tannins that are reported to be responsible for their antidiarrheal activity [37–39].

## 2. Methodology

Databases of Google Scholar, Web of Science, PubMed, Scopus, and SID were searched systematically with no date limitation for English published articles.

The utilized phrases were “phytochemical,” “biological investigations,” “traditional uses,” “pharmacological activity,” “anticancer activity,” “antifungal activity,” “antibacterial activity,” “anti-inflammatory activity,” “medicinal plants,” “antioxidants,” “toxicity,” “commercial products,” “ethnobotany,” and “immunological activity,” with “*Cydonia oblonga*,” or “quince” that reflect subjects of interest up to Feb 2022.

## 3. Results

**Figure 1:**
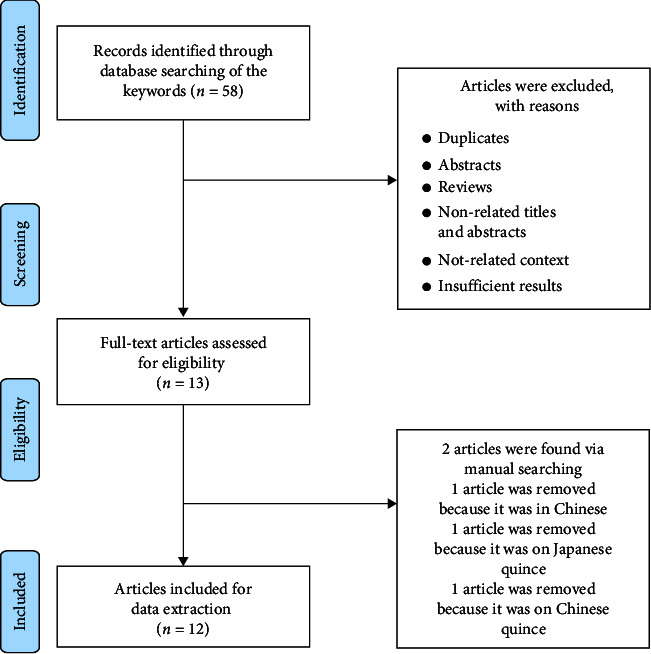
Flowchart of study selection.

Figure 1 shows the selection process. Of 58 articles, 45 were excluded due to duplications and unrelated content/abstract/topic. In among 13 continuous papers, 2 were excluded because they were on Japanese quince [40] and Chinese quince [41]. Two studies were found through manual searching, and finally, 12 were included in our study [12, 22, 42–52]. No human experimental or clinical trial was found, and all studies were on animal or in *vitro* models.  Table 1 listed the details of the included paper.

## 4. Discussion

In 2016, an evaluation of quince's possible bioactive components and nutritional applications was published. We discuss and update the cardiovascular effects of quince, as well as its botany, phytochemistry, and pharmacology, in this review. Reviewing of all 13 included articles on this topic showed that different types of quince extract have positive and beneficial effects on cardiovascular-related factors such as blood pressure [50, 52, 53], diabetes, and glucose metabolism but not on the insulin level [22, 47, 48], lipid profile [12, 43–46, 49], serum biomarkers of liver function [12, 43, 44, 46], body weight [45], and thrombosis [51]. The quince leaf extract's effect on lipid profile, liver enzymes, and atherosclerotic plaque formation in the coronary artery was not significantly different from effects reported in the atorvastatin group [43]. The antihypertensive effect of 320 mg/kg per day of quince leaf extract reported to be not very different from using captopril [52]. None of the studies reported a negative or no positive effect of quince consumption on cardiac-related factors.

### 4.1. Lipid Profile and Body Weight

Of six studies that reported outcomes related to the effect of quince on the lipid profile, all reported significant positive improvement [12, 43–46, 49]. Umar et al. showed that total flavonoids of quince fruit and leaves could adjust the lipid profile in hyperlipidemic rats significantly [49]. In their study high dose of COM total leave flavonoids (160 mg/kg), had lipid-lowering effect almost same as 5 mg/day of simvastatin [49]. According to Khademi et al., the quince group had significantly decreased TC, TG, and LDL_C levels compared to the high-cholesterol diet group (*P* < 0.05), and it was more similar to the normal diet group. Their results showed that quince leaf extract, such as atorvastatin, can successfully adjust the lipid profile; however, both cannot improve atherosclerosis caused on by a high-fat diet significantly [43]. Another study also reported that the aqueous extract of quince fruit significantly reduced serum TG, TC, and LDL-C levels while increasing HDL-C levels in diabetic rats [46]. Abliz et al. found that in hyperlipidemia rats, COM lowered TC, TG, and LDL-C while increasing HDL-C, which was significant at high and medium doses [12]. Lee et al. reported that COM has lipid-adjusting and antiobesity effects, and it can reduce fat mass and adipose tissue weight [45].

### 4.2. Diabetes, Glucose Metabolism, and the Insulin Level

Of five studies that reported outcomes related to the effect of quince on glucose metabolism and diabetes, all reported significant positive improvement [22, 40, 41, 47, 48]. Tang et al. using the *in vitro* model showed that by stimulating the PI3K/AKT signaling pathway, COM prompted glucose metabolism [48]. However, in terms of the insulin level, results were inconsistent, and procyanidin-rich extract of Chinese quince fruit showed no positive effect on the insulin level, while elevation of adiponectin in obese mice resulted in insulin level elevation [45].

### 4.3. Blood Pressure

Of two studies that investigated the effect of COM on blood pressure, both reported significant positive results [50, 52]. It has been shown that extracts of COM leaves (80, 160, and 320 mg/kg) at the highest doses have an antihypertensive effect comparable to captopril [52, 53]. Another study using aqueous-methanolic extract from seeds of quince (200, 400, and 600 mg/kg doses) showed that extract in 600 mg/kg dose produced the most effect and avoided a rise in blood pressure [50].

### 4.4. Oxidative Stress

Oxidative stress is defined as increased intracellular levels of reactive oxygen species (ROS). ROS plays a role in the onset and development of coronary artery disease [54]. ROS is involved in the development of oxidized LDL, which is the first stage in the pathogenesis of atherosclerosis. Furthermore, ROS may activate matrix metalloproteinases, leading to plaque rupture. However, ROS appears to have a significant function in the setting of acute MI and after reperfusion treatment [55]. ROS may oxidize myofibrillar proteins, causing contractile dysfunction in heart failure patients [56]. An in *vitro* study on a polyphenolic extract of Japanese quince fruit showed that it can reduce ROS intracellular [40]. HPLC analysis of the phenolic profile of COM showed that chlorogenic acid was the major phenolic compound in quince leaf extract [19, 45]. Chlorogenic acid is a bioactive polyphenol with potent antioxidant properties which have a heart-protective capacity [57]. Quince fruit had a lot of quinic acid and shikimic derivatives which can protect against cardiovascular disease and cancer [58], as well as procyanidins and flavonoids in it. The presence of 4-caffeoyl shik, which has antioxidative activity, in quince is significantly higher than that of an apple [57].

Quince leaves had a much stronger lowering power than green tea in a comparison research study [19]. In a similar way to green tea, the methanolic extract of quince leaves strongly protected the erythrocyte membrane against hemolysis [19]. The phenolic fraction of quince has been found to have stronger antioxidant activity than the entire methanolic extract [59]. According to Silva et al., organic acid extracts consistently had the lowest antiradical activity, showing that the phenolic component of quince fruit contributes significantly to its antioxidant ability [59]; however, Khademi et al. mentioned that the antioxidant properties of the quince leaf are most likely to be responsible for its antiatherosclerotic effects [43]. In study by Silva et al., methanolic extracts were tested for the antioxidant capacity, and peel extract was shown to have the highest antioxidant activity [59].

A study by Magalhães et al. reported that the DPPH free radical scavenging capabilities of pulp and peel extracts were comparable, whereas seed extract had a substantially lower antioxidant capacity. Pulp and peel extracts significantly protected the erythrocyte membrane from hemolysis under the oxidative action of AAPH in a time-dependent and concentration-dependent manner. Seed extracts alone caused significant hemolysis [60]. These findings imply that the quince leaf or fruit could be employed as a prophylactic or therapeutic agent in free radical-related diseases such as CVD.

Doxorubicin is one of the most regularly used medications to treat a wide range of cancers, and its most common adverse effects include anemia and cardiovascular toxicity. Doxorubicin increases malondialdehyde (MDA) while decreasing glutathione (GSH) and catalase activity in rat heart tissue. Two weeks of treatment with *Cydonia* hydroalcoholic extract significantly reduced MDA levels while raising GSH, demonstrating that Cydonia can relieve oxidative stress and minimize cardiac toxicity [61].

### 4.5. Inflammation

Zhou et al. in their study besides showing the significant antihypertensive effect of quince leaf extract also reported a considerable decrease in the contents of IL-6, IL-1*β*, TNF-*α*, and CRP levels in total flavonoids of COM leaves [53]. It has been documented that chronic inflammation is a hallmark of atherosclerosis [23]. According to a study on a polyphenolic extract from Tunisian COM, it can prevent high levels of the proinflammatory cytokine TNF-, chemokine IL-8, and LPS-mediated activation of three main cellular proinflammatory effectors; p38MAPK, nuclear factor-kappa B, and Akt [23]. Reduced anti-inflammatory cytokine levels, such as CRP, together with lower LDL-C and TC levels, inhibited the development of atherosclerosis [62].

### 4.6. Other Factors

In a study on male rabbits, Khademi et al. discovered that high-cholesterol diet significantly elevated alkaline phosphatase (ALP), ALT, AST, Cr, and BUN. Biochemical markers; AST, ALT, and Cr levels were dramatically reduced when quince leaf extract (50 mg/kg) and atorvastatin 0.5 mg/kg were used in comparison to the control group; however, the difference was not significant [43]. Other studies also proved the positive effect of *Cydonia* on liver function biomarkers (ALP, AST, and ALT) and kidney function biomarkers (Cr and BUN) [44, 46, 49]. However, histological analysis in their study revealed that atorvastatin and COM leaf extract were unable to prevent plaque accumulation in the coronary artery following plaque formation for 12 weeks.

Zhou et al. found that in comparison to aspirin (2.58), extract from *Cydonia* (20, 40, 80 mg/kg/day) prolonged bleeding times by 2.17, 2.78, and 3.63 and the clotting time by 1.44, 2.47, and 2.48 times, respectively [51]. *Cydonia* extract also reduced pulmonary emboli mortality, increased thrombolysis, decreased euglobulin lysis time, and increased the length of venous occlusion in rats compared to the aspirin group [51].

## 5. Conclusion

It can be concluded that *Cydonia oblonga* Miller (quince) has cardiovascular preventive properties. In animal studies, it regulates blood lipids, lowers blood pressure and glucose levels, and protects the liver and kidneys. It also helps with thrombosis and body weight and has anti-inflammatory and antioxidant properties.

## Figures and Tables

**Table 1:** Included studies on *Cydonia oblonga* Miller.

Authors, Year	Model	Intervention	Duration	Studied factors	Results
Zhou et al. [52]	Renal hypertensive rat	Captopril administration 25 mg/kg/day Various doses of COM leaf extracts	8 weeks	SBP&DBP	Blood pressure was considerably and dose-dependently lowered by COM leaf extracts. The greatest. COM dose produced the same results as captopril.
Abliz et al. [12]	Sprague–Dawley rats received high-fat diet	Simvastatin administration Various doses of *COM* leaf extracts	56 days	Lipid profile Liver enzymes MDA Liver steatosis	COM reduced TC, TG, LDL-C and increased HDL-C dose-dependently. COM inhibited the ALT, AST enzymes activity, and reduced liver steatosis.
Zhou et al. [51]	Mice	Aspirin 5 mg/kg/day COM aqueous extracts 20, 40, and 80 mg/kg/day	2 weeks	Bleeding and clotting times	In comparison to aspirin, COM extracts prolonged bleeding and clotting times in a dose-dependent manner.
Khademi et al. [43]	Rabbits	Atorvastatin (0.5 mg/kg) COM leaf extract (50 mg/kg)	12 weeks	Biopsy of coronary artery Lipid profile, AST, ALT, AP, BUN, Cr	Treatment with the COM extract at 50 mg/kg and 0.5 mg/kg atorvastatin lowered plasma lipid profile (*P* 0.05), liver enzymes, BUN, and Cr levels while also increasing HDL.
Khademi et al. [44]	Hypercholsterolemic rabbits	Atorvastatin (0.5 mg/kg) COM leaf extract (50 mg/kg)	8 weeks	Lipid profileLiver enzymes	Treatment with the COM extract at 50 mg/kg and 0.5 mg/kg atorvastatin lowered plasma lipid profile (*P* 0.05), liver enzymes while also increasing HDL-cholesterol levels.
Mirmohammadlu et al. [46]	Diabetic rats	Various doses of aqueous extract of COM fruit		Lipid profile, AST, ALT, AP, BUN, Cr	LDL, TC, TG were significantly decreased. HDL was significantly increased. ALT, AST and ALP were significantly reduced.
Aslan et al. [22]	Healthy and diabetic rats	Various doses of COM leaf extract	5 days	Fasting blood glucose	Blood glucose levels dropped by 33.8 percent
Tang et al. [48]	L6 skeletal muscle cells	Various doses of COM seed extract	1 h	Glucose consumption and glycogen synthesis	At 12.5 g/ml, glucose intake and glycogen production was boosted
Umar et al. [49]	Hyperlipidaemic rats	Various doses of total leaves COM flavonoids Various doses of total fruit COM flavonoids Simvastatin (5 mg/kg)	4 weeks	Lipid profile, Liver enzymes	Total flavonoids from COM fruit and leaves dramatically lowered TC, TG, LDL, ALT, AST, and MDA while considerably increasing HDL.
Mohebbi et al. [47]	Diabetic rats	Various doses of COM fruit aqueous extract	28 days	Fasting blood glucose	A significant collapse in FBG in a dose-dependent manner
Ur rahman et al. [50]	Normotensive, high cholesterol, and glucose fed hypertensive rats	COM extract in 200, 400, and 600 mg/kg dose	21 days	SBP, DBP	SBP decreased significantly in a dose-dependent manner. COM extract in 600 mg/kg dose produced maximum effect and prevented a rise in blood pressure.
Lee et al. [45]	High-fat diet (HFD)-induced obese C57BL/6 mice	HFD + 50 mg/kg BW/day COM (COM 50) HFD + 100 mg/kg BW/day COM (COM 100) HFD + 200 mg/kg BW/day COM (COM 200)	8 weeks	Body weight, fat mass, adipose tissue weight, lipid profile, and leptin	COM showed an anti-obesity effect. COM improved blood HDL, TG, adiponectin levels, body weight, fat mass, adipose tissue weight, insulin resistance, and leptin levels.

COM: *Cydonia oblonga* Miller, ALT: alanine transaminase, AST: aspartate transaminase, ALP: alkaline phosphatase, FBG: fasting blood glucose, SBP: systolic blood pressure, DBP: diastolic blood pressure, TC: total cholesterol, LDL: low-density lipoprotein, HDL: high-density lipoprotein, TG: triglycerides.

## Data Availability

No data were used to support the findings of the study.
